# COVID-19 Outbreak, Mitigation, and Governance in High Prevalent Countries

**DOI:** 10.5334/aogh.3011

**Published:** 2020-09-17

**Authors:** Lung-Chang Chien, Ro-Ting Lin

**Affiliations:** 1 Department of Epidemiology and Biostatistics, University of Nevada, Las Vegas, School of Public Health, Las Vegas, NV, US; 2 Department of Occupational Safety and Health, College of Public Health, China Medical University, Taichung, TW

## Abstract

**Background:**

Disease control involves multiple actions overtime to halt the spread of COVID-19. The role of a country’s governance in slowing the spread of COVID-19 has not yet been well investigated.

**Objective:**

This study aims to investigate the association between governance and the trend of COVID-19 incidence in countries with the highest prevalence. We hypothesized that countries with better governance are more likely to mitigate the spread of COVID-19 than countries with worse governance.

**Methods:**

We analyzed 62 most prevalent countries with at least 10,000 accumulative confirmed cases from January 22 to June 15, 2020. Countries were further grouped into three different levels of governance (25 better governance, 24 fair governance, and 13 worse governance), identified outbreak and mitigation periods using the joinpoint regression model, and compared the number of days and average daily percent change in incidence in two periods by governance level using the one-way analysis of variance.

**Findings:**

The average outbreak period in the 62 countries lasted 84.0 days. Sixty percent of countries (N = 37) had experienced outbreak periods, followed by a mitigation period. In contrast, the rest forty percent of countries (N = 25) still had a rising trend. In the outbreak period, better governance countries had a more rapid increase but a shorter outbreak period (71.2 days) than countries with fair (93.5 days) and worse (90.8 days) governance. Most countries with better governance (84.0%) revealed a declining trend in COVID-19 incidence, while such a trend was less than half of fair and worse governance countries (38.5%–41.7%).

**Conclusions:**

Countries with better governance are more resilient during the COVID-19 crisis. While the mitigation of COVID-19 is observed in most better governance countries, the incidence of COVID-19 is still surging in most fair and worse governance countries, and the possibility of a recurring epidemic of COVID-19 in countries cannot be ignored.

## Introduction

The coronavirus disease 2019 (COVID-19) has been spreading globally for more than five months, since the World Health Organization officially and systematically began reporting the number of confirmed cases of COVID-19 in January 2020 [1]. Disease control involves multiple actions overtime to halt the spread of COVID-19. Scientists are devoted to investigating its pathology to develop treatments and vaccines [2,3], and experts have noted that efficient medical solutions may not be available soon [4,5]. Preventive actions (e.g., limiting international travels, maintaining personal hygiene, wearing masks, social distancing, and staying in lockdown) have therefore been a predominant theme in recent global scientific research to prevent further spreading of COVID-19.

Governance represents the characteristics by which a country manages its authority [6]. It covers the process of selecting, monitoring, and replacing governments, the capability of effectively formulating and implementing sound policies, and the respect of citizens and the country for the institutions that manage their economic and social interactions [6]. Governance influences the design and implementation of health-related policies and services by mobilizing and coordinating stakeholders to realize common goals [7]. Good governance leads to good public health policies and actions [8], which may ultimately contribute to population health. To measure the level of governance across countries, the World Bank’s Worldwide Governance Indicators project captures six dimensions of governance: perceptions of voice and accountability, political stability and absence of violence, government effectiveness, regulatory quality, rule of law, and control of corruption [6]. The perception-based data were used to reflect common views regarding governance outcomes from diverse survey respondents and experts in the surveys of individuals, households, firms, commercial businesses, non-governmental organizations, and public sectors [6]. These six World Bank’s Governance Indicators have been used individually or as a whole to assess their associations with health outcomes in cross-country comparison studies, such as countries with low regulatory quality showed 12 times higher maternal mortality risk is 12 times than in those with high regulatory quality across 174 countries [9], and countries with lower scores of political stability and absence of violence were associated with higher inequality in the coverage of health interventions in the study of 80 low- and middle-income countries [10]. Using this data set of governance indicators, we found that governance is just as important as disease control measures (e.g., immunization and hygiene) in reducing a country’s child mortality [11]. Despite the critical role of governance in controlling the spread of disease, it has not yet been well investigated in the context of COVID-19. Different countries involve mixed aspects of governance. The question is: Would different models of governance that drive collective actions in response to the COVID-19 epidemic lead to different consequences?

Preventive actions proposed by country governments have determined the speed and duration of the spread of the epidemic [12]. Proposing preventive measures is one thing, but implementing them is more critical in determining whether these preventive actions can be successfully carried out, especially when excessive use of force and restrictive measures impose human rights concerns. As COVID-19 cases surged during the early stages of the epidemic, some countries adopted extreme measures to curb the spread of the disease, raising human rights concerns [13,14]. The United Nations Office of the High Commissioner for Human Rights urges countries to pay attention to respecting human rights and protecting vulnerable people, as these are fundamental factors to the success of the public health response and recovery from the COVID-19 pandemic in the long run [15,16]. Responsible governance represents a responsive government [17], especially in more democratic countries where citizens are empowered to voice their needs, participate in public affairs, and request the government’s responsiveness.

Elucidating COVID-19 outbreak information and trends requires the consideration of transparency and corruption, particularly in the context of international politics. Most countries have suffered during the current COVID-19 pandemic, and we hypothesized that countries with better governance are more likely to mitigate the spread of COVID-19 than countries with worse governance. This study applied a time series model to analyze the trend of COVID-19 incidence in countries with the highest prevalence from January 22, 2020 to June 15, 2020. Countries were further grouped into three different levels of governance, and we compared the time to mitigate the outbreak among these three groups.

## Methods

### Data sources and variable definitions

We adopted six governance indicators in 2018 for each country from the Worldwide Governance Indicator published by the World Bank [18]. The six indicators represent the following dimensions of a country’s governance: (i) voice and accountability, (ii) political stability and absence of violence/terrorism, (iii) government effectiveness, (iv) regulatory quality, (v) the rule of law, and (vi) control of corruption [6]. Supplementary Table S1 defines each of these indicators. For cross-country comparison, scores of each indicator have been transferred to the normal distribution (mean = 0 and standard deviation = 1), ranging approximately from –2.5 to 2.5. Higher scores represent better governance.

We obtained the daily accumulative confirmed cases of COVID-19 between January 22, 2020, and June 15, 2020 at the country level from the Johns Hopkins Coronavirus Resource Center [19], and then calculated the daily new cases accordingly. We further calculated the 7-day moving average of daily new confirmed cases in each country. The estimated population data of 2020 by country were also derived from the Worldometer database to calculate the incidence rate per 1 million people [20]. Notice that only countries with at least 10,000 accumulative confirmed cases through June 15 were included in our samples for further analyses.

### Statistical analyses

To assess the level of governance, we first applied a cluster analysis to group 166 countries according to a similarity measure derived from the mean absolute deviation of the six governance indicators. The criterion in determining the number of clusters was the R [2] of 0.7, resulting in three clusters, with 35.5% (N = 59), 36.2% (N = 60), and 28.3% (N = 47) countries in each cluster (see Supplementary Figure S1).

We used the joinpoint regression model to analyze the trend of COVID-19 incidence and to detect whether an apparent downward trend had happened in each selected country [21]. Unlike the nonlinear model, which can address the detailed variation of a trend, we alternatively aim to fit the trend by combining several straight lines to explain the daily change as the general linear model. That is the rationale of choosing the joinpoint regression model because it can efficiently evaluate a trend by connecting several lines with joinpoints, which depict time points significantly changing from downward to upward and vice versa. Because all lines are based on the log-linear model, the joinpoint regression model is free from complex spline selections and sensitivity concerns. This modeling approach first used a grid method to scan the whole trend to find out possible joinpoints where significant changes occurred over time. We considered at most five joinpoints in each country. A model selection was used to choose how many joinpoints were most appropriate, according to the smallest Bayesian information criteria. When *j* (*j* ≤ 5) joinpoints were determined, the whole trend was partitioned into (*j* + 1) segments, and the change during each time segment was estimated by the following equation:

{\log} (case) = \alpha + \beta \times t + {\log}(population)

where *t* is a calendar time variable from the date of the first confirmed case (*t* = 1) until June 15. If a country has a peak joinpoint with the highest modeled incidence rate, the identified peak joinpoint was defined as a threshold to split the entire trend into an outbreak period from *t* = 1 to the time of the identified peak joinpoint and a mitigation period from the time of the identified peak joinpoint to June 15. The number of days and the average daily percent change (ADPC) in each period were calculated [22]. The two metrics were further compared among the three governance clusters by using the one-way analysis of variance and the post-hoc test with Tukey’s adjustment, respectively.

Data cleaning and management were done by SAS v9.4 (SAS Institute Inc., Cary, NC). Data analyses were implemented by Joinpoint Regression Software v.4.8.0.1 (National Cancer Institute, Bethesda, MD). The significance level was set to 0.05.

## Results

By June 15, the number of cases in the 62 high prevalent countries was 7.80 million, accounting for 97.2% of the total confirmed cases in the world. Among the 62 countries, sixty of countries (N = 37) had experienced outbreak periods, followed by a mitigation period. In contrast, the rest forty percent of countries (N = 25) still had a rising trend through June 15. Table 1 shows the number of days and ADPC at the country level during the outbreak period (i.e., before the identified peak joinpoint) and the mitigation period (i.e., after the identified peak joinpoint). The average outbreak period in the 62 countries lasted 84.0 days, with an ADPC of 11.5%. Among 37 countries with the mitigation period, the average mitigation period has lasted 54.9 days, with an ADPC of –2.5%.

**Table 1:** The number of days and average daily percent change during the outbreak period and the mitigation period, by country.

Rank	Country	Cluster	Whole period	Outbreak period		Mitigation period	

			# Days	# Days	ADPC	# Days	ADPC

*1*	United States	1	146	77	29.27	70	–0.60
*2*	Brazil	2	111	111	11.70	–	–
*3*	Russia	2	137	103	13.24	35	–0.73
*4*	India	2	138	138	10.32	–	–
*5*	United Kingdom	1	137	72	16.37	66	–2.20
*6*	Spain	1	136	60	29.58	77	–4.25
*7*	Italy	1	137	56	18.47	82	–3.67
*8*	Peru	2	102	87	9.84	16	–2.82
*9*	France	1	144	86	12.01	59	–5.10
*10*	Iran	3	118	43	16.03	76	–0.47
*11*	Germany	1	141	70	12.40	72	–4.26
*12*	Turkey	3	97	33	25.68	65	–2.06
*13*	Chile	1	105	105	8.06	–	–
*14*	Mexico	2	109	109	8.61	–	–
*15*	Pakistan	3	112	112	9.81	–	–
*16*	Saudi Arabia	2	106	106	8.23	–	–
*17*	Canada	1	142	92	9.00	51	–2.76
*18*	Bangladesh	3	100	100	8.04	–	–
*19*	China	2	146	29	7.65	118	–5.27
*20*	Qatar	1	108	92	8.88	17	–1.53
*21*	South Africa	2	103	103	8.92	–	–
*22*	Belgium	1	133	72	14.36	62	–4.31
*23*	Belarus	2	109	82	9.17	28	–0.75
*24*	Colombia	2	102	102	8.42	–	–
*25*	Sweden	1	137	137	5.60	–	–
*26*	Netherlands	1	110	55	11.09	56	–3.26
*27*	Ecuador	3	107	55	10.26	53	–2.97
*28*	Egypt	3	123	123	6.27	–	–
*29*	United Arab Emirates	1	139	118	4.76	22	–2.73
*30*	Singapore	1	145	93	8.23	53	–2.03
*31*	Indonesia	2	106	106	6.96	–	–
*32*	Portugal	1	106	32	23.39	75	–1.24
*33*	Kuwait	2	113	83	5.82	31	–1.64
*34*	Argentina	2	105	105	7.68	–	–
*35*	Ukraine	3	105	105	8.60	–	–
*36*	Switzerland	1	112	30	25.25	83	–4.88
*37*	Poland	1	104	104	6.10	–	
*38*	Philippines	2	138	123	5.53	16	–0.55
*39*	Afghanistan	3	113	103	8.66	11	–1.34
*40*	Ireland	1	108	47	17.70	62	–6.68
*41*	Oman	1	113	113	6.98	–	–
*42*	Dominican Republic	2	107	107	8.56	–	–
*43*	Romania	2	111	44	16.68	68	–0.74
*44*	Panama	2	98	98	5.76	–	–
*45*	Iraq	3	113	113	5.85	–	–
*46*	Israel	1	116	40	21.95	77	–1.49
*47*	Bolivia	3	97	97	6.42	–	–
*48*	Bahrain	2	113	113	3.80	–	–
*49*	Japan	1	146	82	7.18	65	–3.75
*50*	Austria	1	112	32	26.34	81	–4.14
*51*	Armenia	2	107	99	5.96	9	–0.41
*52*	Nigeria	3	109	109	7.52	–	–
*53*	Kazakhstan	2	95	83	4.80	13	–1.29
*54*	Denmark	1	110	42	20.21	69	–3.68
*55*	Serbia	2	102	45	12.22	58	–2.72
*56*	South Korea	1	146	42	23.52	105	–2.34
*57*	Ghana	2	94	62	7.61	33	–0.52
*58*	Moldova	2	100	100	5.84	–	–
*59*	Algeria	3	112	93	6.86	20	–2.86
*60*	Azerbaijan	2	107	107	6.19	–	–
*61*	Guatemala	3	94	94	6.89	–	–
*62*	Czech	1	107	31	20.26	77	–2.13

Abbreviation: # Days = number of days; ADPC = average daily percent change. Cluster 1 indicates better governance, Cluster 2 indicates fair governance, and Cluster 3 indicates worse governance.

**Figure 1:**
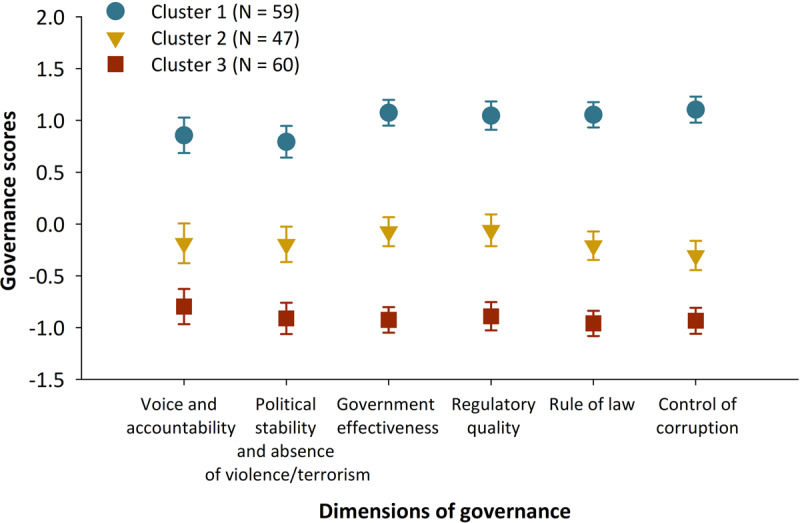
Least squares means and 95% confidence intervals of six dimensions of governance among three clusters of 166 countries. Abbreviation: N = number of countries.

Figure 1 displays the least square means of three clusters of countries by the six governance dimensions. The one-way analysis of variance shows that all governance indicators were significantly different among the three clusters (all p-values < 0.001). Cluster 1 had the highest least square means for all governance indicators, indicating that countries in this group had better governance. Cluster 3 had the lowest least square means for all governance indicators, indicating worse governance. We defined the countries in cluster 2 as fair governance countries. Among the selected 62 countries, 40.3% of countries (N = 25) are in cluster 1 (better governance), 38.7% (N = 24) in cluster 2 (fair governance), and 21.0% (N = 13) in cluster 3 (worse governance).

**Figure 2:**
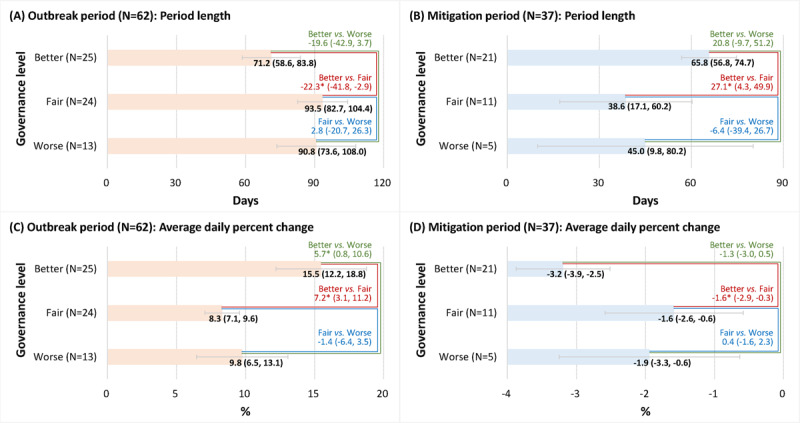
Means of period length and average daily percent change during the outbreak period and the mitigation period by governance cluster, and pairwise comparisons among governance clusters. The 95% confidence intervals were shown in parentheses. * p < 0.05.

Significant differences in the number of days and ADPC during the two periods were found among the three governance clusters (Figure 2). In the outbreak period, better governance countries averagely spent 71.2 days to reach the peak, which was 19.6–22.3 days shorter than fair and worse governance countries. In the mitigation period, better governance countries have been lasting for 65.8 days, longer than fair and worse governance countries by 20.8–27.1 days. By comparing with the ADPC of fair and worse governance countries, the better governance countries experienced a more rapid increase in the outbreak period (ADPC = 15.5%; 5.7%–7.2% faster than in fair and worse governance countries) and decreased in the mitigation period (ADPC = –3.2%; 1.3%–1.6% faster than in fair and worse governance countries).

Among 25 countries with a rising trend through June 15, 52.0% (N = 13) of them were fair governance countries, and 32.0% (N = 8) of them were worse governance countries. Only four countries (Chile, Sweden, Poland, and Oman) were in better governance. The average of ADPC was 6.7%, 7.8%, and 7.4% in better, fair, and worse governance countries, respectively. No significant difference was identified among the three clusters.

## Discussion

National governance plays a critical role in determining how a country copes with the fast-paced dynamic of COVID-19 [23]. Our findings indicate that countries with better governance are more resilient. Although better governance countries experience rapid surges in the number of cases during the COVID-19 crisis, the incidence decreases steadily. Because COVID-19 has spread rapidly since February 2020, a country’s first step is to identify sources of the virus and diagnose infected cases. Understanding the routes and timing of transmission helps governments shape and implement effective prevention measures [12]. Governments need sufficient medical capacity for screening and may need to ask employers to give workers sufficient leave time for disease prevention and to relieve the public’s worries. If more people are willing to disclose their illness to public health authorities, the increasing number of confirmed cases should be no surprise when a country’s disease surveillance system works. For instance, the rapid and large-scale screening strategy in South Korea demonstrates its capability to diagnose COVID-19, including sufficient laboratory and medical resources, government policies, and the capacity to mobilize and authorize resources to both public and private hospitals and laboratories across the country. For instance, the Korean Food and Drug Administration shortened the process for the approval of new test kits [24]. To engender public trust and avoid unreasonable public panic and confusion during the COVID-19 crisis, governments should also be timely and transparent in disclosing information, such as the number of cases, suspected sources of exposure, and what actions have been performed [25]. Overall, a better capacity in capturing and reporting disease outbreaks can be expected in countries with better governance.

Our results show the outbreak period in better governance countries was 19.6–22.3 days shorter than those in fair and worse governance countries. Given the necessity for a rapid response to this once-in-a-century global pandemic, the government’s next step should be solving the problem and mitigating the disease burden. Relative to countries with worse governance, we found stronger evidence of steady declines in the incidence of COVID-19 among countries with better governance. We hypothesized that the control of COVID-19 is faster in countries characterized as having better governance. Rapid and intensive public health measures, from personal protective measures (e.g., hand hygiene and masks) to large-scale restrictive public health measures (e.g., lockdown and quarantining contacts), should be implemented to slow the spread of COVID-19 and stop transmission [26]. These collective measures to fight against COVID-19 for the sake of public health are regarded as public goods [27]. Harsh steps may be options, but without good governance, COVID-19 may turn back and even lead to governments violating human rights. Efforts to detect, prevent, and treat COVID-19 must be sustained, including after therapeutics and vaccines are successfully developed and manufactured [28]. Good governance is the foundation needed to link existing systems and ensure collective measures are implemented and equally distributed, as well as return to normal life in the long term [26,28].

Austria is another good example of a country with better governance and controlled the outbreak. The number of days in the outbreak period was 32 days in Austria. On March 9, 2020, immediately after COVID-19 struck Europe, the Austrian government introduced short-term restrictive measures (e.g., banned gatherings, border controls, social distancing and self-isolation, compulsory face masks in public areas), and a month later, it announced a step-by-step timetable to ease the lockdown and revive the economy [29]. The country has sustained a low incidence of almost three months. Eventually, different governance models should be associated with COVID-19 epidemic trends. Continuous regulation and provision of resources to support society, industry, and research can improve disease control and keep the number of cases low, ameliorate the disease burden, and prevent future outbreaks.

The authors acknowledge the following limitations to this study. First, the case definition of COVID-19 varies among countries. A recent study revealed that China has had several versions of the case definition for COVID-19, and if the fifth version of the definition—a new category of cases for Hubei province named “clinically diagnosed case,” which was defined as a suspected case with pneumonia indicated by chest radiograph but didn’t require a virological confirmation of infection—had been applied throughout the whole outbreak, the total number of cases would increase over 400% [30]. We are unable to know whether other countries have experienced the same situation. Second, our findings have a strong assumption that all confirmed cases were infected on the same day; nonetheless, there might be a lagged effect between the true infected date and the diagnosed date. Reasons for lagged diagnoses could include insufficient sieving reagents at the beginning of outbreaks or asymptomatic cases [31,32]. Third, we included only those countries with at least 10,000 accumulative confirmed cases in our analysis. Estimates should be cautiously interpreted and may not be guaranteed in countries not included. However, that should not preclude us from sharing the experience of some good governance countries. A recent study highlighted high transmissibility of COVID-19 even before symptom onset [33]. While asymptomatic transmission will become a more important source, wearing masks may have played a substantial protective role [34,35]. The immediate question is how the government takes this preventive action to ensure universal coverage and equal access. One example of a country that demonstrated rapid collaboration between ministries and public-private partnerships is Taiwan, which is geographically and culturally similar to China but still managed to keep the number of COVID-19 cases low [36,37]. Three days after the first imported case on January 21, 2020, Taiwan implemented an export ban on medical and N95 masks to secure domestic use and established a national team to boost mask production. More importantly, the government initiated a name-based rationing system for mask purchases to ensure equal access to quality-assured masks for everyone and to cope with the general public’s fears. The case of Taiwan demonstrates that good governance is linked to sound disease prevention policies, responsiveness to people’s needs, and protection of the health of the total population [38].

## Conclusions

The impact of COVID-19 on the health of a country’s population reflects the status quo and resilience of its governance. We found that countries with better governance had a more rapid increase but a shorter outbreak period than countries with fair or worse governance by 19.6–22.3 days. Most countries with better governance (84.0%) revealed a declining trend in COVID-19 incidence, while such a trend was less than half of fair and worse governance countries (38.5%–41.7%). While the mitigation of COVID-19 is observed in most countries with better governance, a hidden worry, however, may affect the pandemic in the near future: the incidence of COVID-19 is still surging in most countries with fair and worse governance, and the possibility of a recurring epidemic of COVID-19 in countries cannot be ignored.

## Additional Files

The additional files for this article can be found as follows:

10.5334/aogh.3011.s1 Supplementary Table S1. Definition of six indicators of governance.

10.5334/aogh.3011.s2 Supplementary Figure S1. Tree plot of cluster analysis according to six governance indicators.
