# Supplementary material for: COVID-19 Outbreak, Mitigation, and Governance in High Prevalent Countries
Source: Ann Glob Health. 2020 Sep 17;86(1):119. doi: 10.5334/aogh.3011 (PMC7500241; doi:10.5334/aogh.3011)
Supplement: Supplementary Table S1. — Definition of six indicators of governance. [file agh-86-1-3011-s1.pdf]

Supplementary Table S1. **Definition of six indicators of governance**

Indicator	Definition
Voice and accountability	The extent to which a country's citizens are able to participate in selecting their government, as well as freedom of expression, freedom of association, and a free media.
Political stability and absence of violence/terrorism	The likelihood of political instability and/or politically motivated violence, including terrorism.
Government effectiveness	The quality of public services, the quality of the civil service and the degree of its independence from political pressures, the quality of policy formulation and implementation, and the credibility of the government's commitment to such policies.
Regulatory quality	The ability of the government to formulate and implement sound policies and regulations that permit and promote private sector development.
Rule of law	The extent to which agents have confidence in and abide by the rules of society, and in particular the quality of contract enforcement, property rights, the police, and the courts, as well as the likelihood of crime and violence.
Control of corruption	The extent to which public power is exercised for private gain, including both petty and grand forms of corruption, as well as "capture" of the state by elites and private interests.

Reference: D. Kaufmann, A. Kraay, M. Mastruzzi, Governance matters VIII : aggregate and individual governance indicators 1996-2008. Policy Research Working Paper (The World Bank, Washington, DC, USA, 2009), vol. No. 4978.
