# Supplementary figures and images for: COVID-19 Outbreak, Mitigation, and Governance in High Prevalent Countries
Source: Ann Glob Health. 2020 Sep 17;86(1):119. doi: 10.5334/aogh.3011 (PMC7500241; doi:10.5334/aogh.3011)

1 **Supplementary Figure S1. Tree plot of cluster analysis according to six governance**  
2 **indicators.**

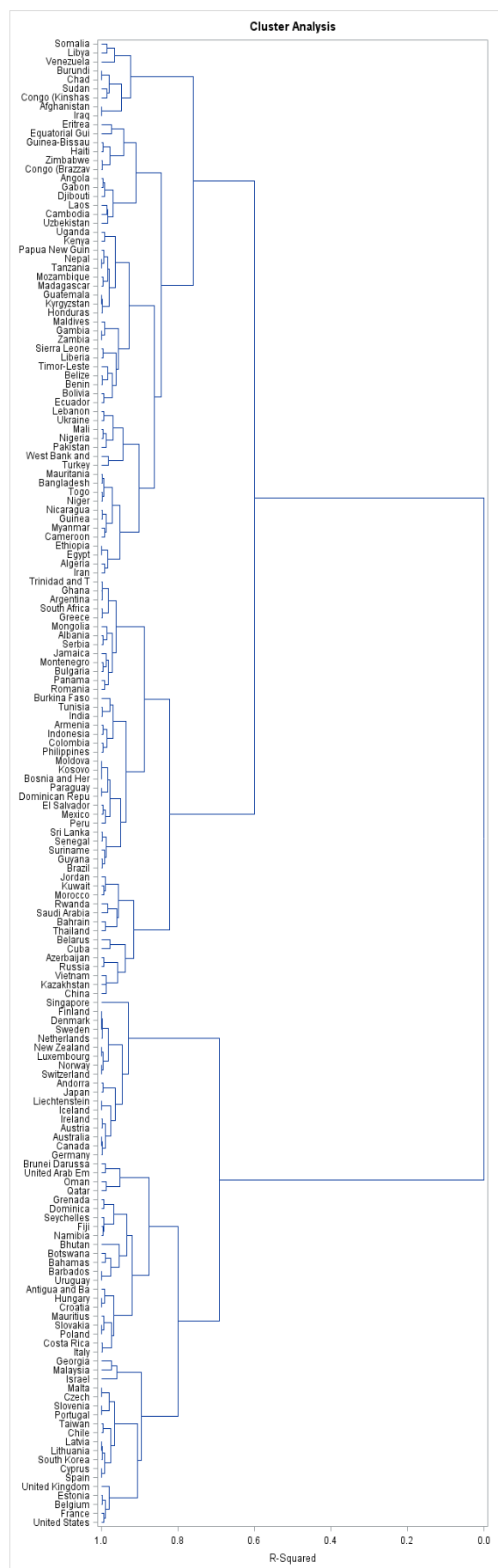

Supplement: Supplementary Figure S1. — Tree plot of cluster analysis according to six governance indicators. [file agh-86-1-3011-s2.pdf]
